# Evidence for the spread of SARS-CoV-2 and olfactory cell lineage impairment in close-contact infection Syrian hamster models

**DOI:** 10.3389/fcimb.2022.1019723

**Published:** 2022-10-21

**Authors:** Rumi Ueha, Toshihiro Ito, Satoshi Ueha, Ryutaro Furukawa, Masahiro Kitabatake, Noriko Ouji-Sageshima, Tsukasa Uranaka, Hirotaka Tanaka, Hironobu Nishijima, Kenji Kondo, Tatsuya Yamasoba

**Affiliations:** ^1^ Swallowing Center, The University of Tokyo Hospital, Tokyo, Japan; ^2^ Department of Otolaryngology and Head and Neck Surgery, Faculty of Medicine, The University of Tokyo, Tokyo, Japan; ^3^ Department of Immunology, Nara Medical University, Nara, Japan; ^4^ Division of Molecular Regulation of Inflammatory and Immune Diseases, Research Institute for Biomedical Sciences, Tokyo University of Science, Chiba, Japan; ^5^ Department of Otorhinolaryngology, The Jikei University School of Medicine, Tokyo, Japan

**Keywords:** SARS-CoV-2, close contact, short-term cohabitation, olfactory receptor cells, nose, lung, olfactory epithelium

## Abstract

**Objectives:**

Close contact with patients with COVID-19 is speculated to be the most common cause of viral transmission, but the pathogenesis of COVID-19 by close contact remains to be elucidated. In addition, despite olfactory impairment being a unique complication of COVID-19, the impact of SARS-CoV-2 on the olfactory cell lineage has not been fully validated. This study aimed to elucidate close-contact viral transmission to the nose and lungs and to investigate the temporal damage in the olfactory receptor neuron (ORN) lineage caused by SARS-CoV-2.

**Methods:**

Syrian hamsters were orally administered SARS-CoV-2 nonvariant nCoV-19/JPN/TY/WK521/2020 as direct-infection models. On day 3 after inoculation, infected and uninfected hamsters were housed in the same cage for 30 minutes. These uninfected hamsters were subsequently assigned to a close-contact group. First, viral presence in the nose and lungs was verified in the infection and close-contact groups at several time points. Next, the impacts on the olfactory epithelium, including olfactory progenitors, immature ORNs, and mature ORNs were examined histologically. Then, the viral transmission status and chronological changes in tissue damage were compared between the direct-infection and close-contact groups.

**Results:**

In the close-contact group, viral presence could not be detected in both the nose and lungs on day 3, and the virus was identified in both tissues on day 7. In the direct-infection group, the viral load was highest in the nose and lungs on day 3, decreased on day 7, and was no longer detectable on day 14. Histologically, in the direct-infection group, mature ORNs were most depleted on day 3 (p <0.001) and showed a recovery trend on day 14, with similar trends for olfactory progenitors and immature ORNs. In the close-contact group, there was no obvious tissue damage on day 3, but on day 7, the number of all ORN lineage cells significantly decreased (p <0.001).

**Conclusion:**

SARS-CoV-2 was transmitted even after brief contact and subsequent olfactory epithelium and lung damage occurred more than 3 days after the trigger of infection. The present study also indicated that SARS-CoV-2 damages all ORN lineage cells, but this damage can begin to recover approximately 14 days post infection.

## Introduction

Coronavirus disease (COVID-19), caused by severe acute respiratory syndrome coronavirus 2 (SARS-CoV-2), has been declared a pandemic since the end of December 2019 (Guan et al., 2020; Castagnoli et al., 2020) that remains to be contained; rather, the infected population is increasing worldwide. The incubation period from SARS-CoV-2 exposure to the onset of COVID-19 symptoms is reported to be approximately 6 days, and the viral load in droplets increases several days prior to the onset of symptoms in patients with COVID-19 (Backer et al., 2020; He et al., 2020; Lauer et al., 2020). Accordingly, if virus carriers do not take appropriate infection control measures during the asymptomatic period, they may play an important role in unintentional COVID-19 spread (Oran and Topol, 2020; Nikolai et al., 2020). The most likely asymptomatic carriers are considered close contacts, defined as individuals who had contact with infected persons for more than 15 minutes within a 1-m distance without properly wearing masks, and these individuals are considered at high risk of exposure to SARS-CoV-2 and developing infection (Organization WH, 2022; Prevention CDC, 2022). Nevertheless, how the virus spreads and multiplies in the body after brief close contact with an infected person has not been sufficiently studied.

The nasal cavity comprises important tissue for the replication of SARS-CoV-2, and SARS-CoV-2 can cause chemosensory dysfunction and affect olfaction (Biadsee et al., 2020; Butowt and von Bartheld, 2021). In the early days of the COVID-19 pandemic, olfactory dysfunction was often the first manifestation of COVID-19 (Saniasiaya et al., 2021). The prevalence and severity of COVID-19-related olfactory dysfunction has decreased since the omicron variant became prevalent, but it remains an important issue as a sequela of COVID-19 (Boscolo-Rizzo et al., 2022). Despite the nose being an essential sensory organ, it has not been fully elucidated whether infection resulting from short-term contact results in damage of nasal epithelium. In previous studies using animal models, animals were administered high doses of SARS-CoV-2, and severe nasal epithelial damage was identified within a few days after viral exposure (Golden et al., 2020; Bryche et al., 2020; Urata et al., 2021; Seo et al., 2021; Kishimoto-Urata et al., 2022). However, as high doses of viruses are unlikely to be taken in at once in a real-life environment, the damage to the olfactory epithelium (OE) of the nose in these SARS-CoV-2 infection models may be discrepant from the actual situation. Therefore, it is imperative to validate close-contact infection models that readily resemble the infection situation in daily life. In addition, SARS-CoV-2 can be transmitted both nasally and orally, and the longitudinal tissue damage after nasal and oral virus administration needs to be evaluated.

The OE is composed of supporting cells (sustentacular cells), basal progenitor cells, immature olfactory receptor cells (ORNs), and mature ORNs (Ueha et al., 2016). Basal cells and supporting cells are particularly susceptible to damage by SARS-CoV-2 infection, and the entire OE may be denuded depending on the site (Butowt and von Bartheld, 2021; Urata et al., 2021; Seo et al., 2021; Ueha et al., 2022). This OE impairment improves over time, and the OE is nearly normalized within approximately 1 month after SARS-CoV-2 infection, although the regenerative kinetics may differ according to the nose area, such as the nasal septum and lateral nasal turbinate (Urata et al., 2021). Although temporal histological changes in the epithelial thickness of the OE over time have been reported, the impact of SARS-CoV-2 on the various cell groups comprising the OE over time has not been verified. As the susceptibility to damage and regeneration progression varies depending on the location in the nose (Tuerdi et al., 2018; Ueha et al., 2020), it would be significant to examine the effects of SARS-CoV-2 on the OE, especially on the ORN lineage, in a site-specific manner. Furthermore, it would be noteworthy to elucidate the impact of SARS-CoV-2 infection in different cell types after transmission by short-term close contact.

In this study, to elucidate the biological effects of short-term close contact with SARS-CoV-2 infected individuals, we created short-term contact models and examined histological and molecular biological changes over time in the nose and lungs. Next, we examined the temporal changes in the OE in a nasal-cavity site-specific manner in hamsters infected with SARS-CoV-2. Last, we compared the time course of virus spread in tissues between direct-infection and close-contact models.

## Materials and methods

### Animals

We selected Syrian hamsters (*Mesocricetus auratus*) as the animal model for this study because Syrian golden hamsters have been used in various studies related to SARS-CoV-2 and are recognized as an excellent small animal model for SARS-CoV-2 infection (Imai et al., 2020; Lee and Lowen, 2021) and also for transmission (Furukawa et al., 2021). Six-week-old, male, Syrian hamsters were purchased from Japan SLC (Hamamatsu, Shizuoka, Japan) and maintained in a specific pathogen-free environment at the Animal Research Center of Nara Medical University. The animal experimental protocols followed the ARRIVE guidelines and were approved by The Animal Care and Use Committee at Nara Medical University (approval number, 12922). All procedures were performed in compliance with relevant guidelines on the Care and Use of Laboratory Animals, Nara Medical University and Animal Research, and Animal Care and Use Committee of the University of Tokyo.

### Animal model preparation

**Figure 1:**
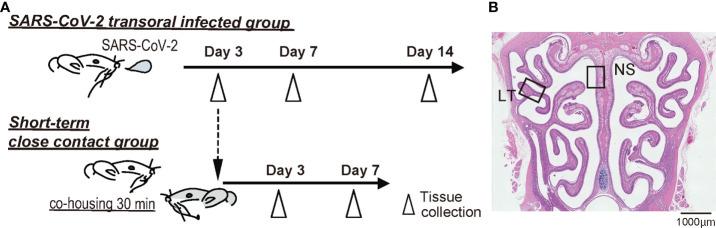
Experimental timeline and nasal structure. **(A)** Hamsters were administered orally severe acute respiratory syndrome coronavirus 2 (SARS-CoV-2) (1.0×10^3^ pfu). To prepare the short-term close-contact models, the uninfected hamsters were cohoused for 30 minutes with SARS-CoV-2 infected hamsters (3 days after SARS-CoV-2 inoculation), and then separated. **(B)** Representative images of the olfactory epithelium from control hamsters. The boxes indicate the regions of the dorsal nasal septum (NS) and lateral turbinate (LT) areas.

SARS-CoV-2 infected animal models were inoculated following previously reported methods (Furukawa et al., 2021; Ueha et al., 2022). In short, the SARS-CoV-2 strain (nonvariant nCoV-19/JPN/TY/WK521/2020, provided by the National Institute of Infectious Diseases, Japan) was used. Virus culture was performed using VeroE6/TMPRSS2 cells (JCRB Cell Bank in Japan, JCRB1819), following which, virus extracts were prepared. The virus stocks were titrated to determine pfu in VeroE6/TMPRSS2 cells using the plaque assay method as previously reported (PMID34880383). Twenty-four hamsters were distributed into three groups: four hamsters in the negative control group, 12 hamsters in the SARS-CoV-2 transoral infection group (infected group), and eight hamsters in the short-term close-contact group (contact group). All experiments using SARS-CoV-2 were performed at the biosafety level 3 experimental facility of Nara Medical University, Japan. The time course is shown in **Figure 1A**.

The hamsters were anesthetized with an intraperitoneal injection of pentobarbital (10 mg/mL, 0.8 mL/hamster) before virus inoculation. To the SARS-CoV-2 transoral infection group, 50 μL of virus solution diluted with saline (including 1.0×10^3^ pfu of SARS-CoV-2) was administered orally as previously reported (Furukawa et al., 2021; Ueha et al., 2022). To the control group, 50 μL of saline was administered. At 3, 7, and 14 days after inoculation, the SARS-CoV-2-inoculated hamsters were euthanized by intraperitoneal injection of 1.0 ml sodium pentobarbital (10 mg/mL) followed by cardiac exsanguination.

To prepare the short-term close-contact models, uninfected hamsters were transferred to the cages of infected hamster on day 3 of SARS-CoV-2 administration and cohoused. After 30 minutes of cohabitation, the short-term close-contact hamsters and directly infected hamsters were separated. Three and 7 days after contact, the close-contact-group hamsters were euthanized. The noses were sampled for histopathological examination, and the lungs were sampled for histopathological examination (the left lobe) and quantitative polymerase chain reaction (qPCR, the right lobe).

### RNA extraction and RT-qPCR

We validated SARS-CoV-2 viral RNA in the lungs to confirm the SARS-CoV-2 infection status of each hamster. Total RNA was isolated from the lung using NucleoSpin^®^ RNA (Macherey-Nagel, Düren, Germany), and then converted to cDNA using a High-Capacity cDNA Reverse Transcription Kit (Thermo Fisher Scientific, Waltham, MA, USA), according to the previous protocol (Furukawa et al., 2021; Ueha et al., 2022) and the manufacturer’s instructions. RT-qPCR analysis was performed using a StepOnePlus Real-Time PCR System (Thermo Fisher Scientific). The gene-specific primers and probes used were: *Gapdh* as endogenous control (TaqMan assay Cg04424038) and SARS-CoV-2 nucleocapsid gene (forward: 5’- AAATTTTGGGGACCAGGAAC -3’, reverse: 5’- TGGCAGCTGTGTAGGTCAAC -3’, the TaqMan probe: FAM-ATGTCGCGCATTGGCATGGA-BHQ). The expression levels of each gene were normalized to the level of *Gapdh* expression for each sample.

### Tissue preparation

Immediately after tissue harvesting, the lungs and nasal tissues assigned for histological analyses were gently irrigated and fixed in 4% paraformaldehyde for 14 days, and the nose samples were decalcified. Then, each sample was dehydrated in graded ethanol solutions, and embedded in paraffin. Regarding the nose tissues, coronal sections were obtained from all samples at the level of the anterior end of the olfactory bulb for histological analysis of the olfactory mucosa (Ueha et al., 2016; Ueha et al., 2016; Ueha et al., 2018; Ueha et al., 2018). Four-micrometer-thick paraffin sections were deparaffinized in xylene and rehydrated in ethanol before staining. Regarding the lung tissues, the left lobe of the lungs was used for histological assessment, and two-micrometer-thick paraffin sections were prepared.

### Histological analyses

Hematoxylin and eosin staining was performed to evaluate the overall tissue structure. For immunostaining, after antigen retrieval, deparaffinized sections were treated with 3% hydrogen peroxide to block endogenous peroxidase activity and then incubated in Blocking One solution (Nacalai Tesque, Kyoto, Japan) to block non-specific immunoglobulin binding. Then, the samples were incubated with primary antibodies, followed by secondary antibodies.

The primary antibodies used in this study are listed in **Table 1**. Anti-SARS-CoV-2 nucleocapsid antibody was used to identify SARS-CoV-2. The following antibodies were used to evaluate ORN neurogenesis: sex-determining region Y-box 2 (SOX2), expressed by proliferating stem cells or progenitor cells in the basal layer of the OE; growth-associated protein 43 (GAP43), expressed by immature ORNs in the OE; and olfactory marker protein (OMP), expressed by mature ORNs in the OE.

**Table 1:** Primary antibodies used in this study.

Primary antibody	Source	Catalog No.	Host	Type	Dilution
SARS-CoV-2 nucleocapsid	GeneTex (Irvine, CA, USA)	GTX135357	Rabbit	polyclonal	1:1000
SOX2	Abcam (Cambridge, UK),	ab92494	Rabbit	monoclonal	1:300
GAP43	Novus Biologicals (Centennial, CO, USA)	NB300-143B	Rabbit	polyclonal	1:1000
OMP	Wako (Tokyo, Japan)	019-22291	Goat	polyclonal	1:8000

The ORNs are classified into four groups according to their zonal-expression patterns, and odorant receptors are expressed by sensory neurons distributed within one of four circumscribed zones (Yoshihara et al., 1997; Mori et al., 2000; Imamura and Hasegawa-Ishii, 2016). Of these, zone 1 is determined by co-localization with NQO1 expression and zones 2–4 are determined by OCAM expression (Yoshihara et al., 1997; Mori et al., 2000; Gussing and Bohm, 2004; Imamura and Hasegawa-Ishii, 2016). Our previous studies have shown that the OE of the dorsal nasal septum area represents OCAM^-^/NQO1^+^ expression (Zone 1) and that of the lateral area represents OCAM^+^/NQO1^-^ expression (zones 2–4). Therefore, to analyze the OE, we divided coronal sections of the OE into two areas along the zonal organization: the dorsal nasal septum (NS) area and lateral turbinate (LT) area (**Figure 1B**).

Images were captured using a digital microscope camera (Keyence BZ-X700, Osaka, Japan) with 10× and 40× objective lenses. OMP^+^ ORNs, SOX2^+^ ORN progenitors, and GAP43^+^ immature ORNs in a 300-μm region of each area were counted in the right and left sides of each sample. The number of each cell type was quantitatively analyzed using sections with immunostaining for each antigen and counterstaining with hematoxylin.

### Statistical analysis

Statistical comparisons between groups were performed with one-way analysis of variance using GraphPad Prism software (version 6.7; GraphPad Software, Inc., San Diego, CA, USA, www.graphpad.com). qPCR data were subjected to logarithmic transformation prior to analysis. Results with *p* < 0.05 were considered statistically significant.

## Results

### Time course of SARS-CoV-2 infection after oral virus inoculation is analogous in the nose and lungs with a tendency to subside 7 days post infection

**Figure 2:**
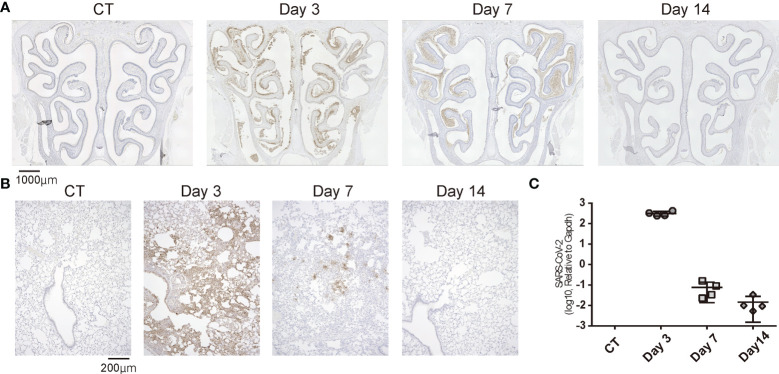
Temporal SARS-CoV-2 infection findings in the nose and lungs. **(A)** Representative images of immunohistological staining of severe acute respiratory syndrome coronavirus 2 (SARS-CoV-2) in control (CT) hamsters and SARS-CoV-2 hamsters on days 3, 7, and 14. SARS-CoV-2 is shown in brown. **(B)** Immunohistochemistry staining of SARS-CoV-2 in the lung. **(C)**: SARS-CoV-2 gene detection with RT- quantitative polymerase chain reaction in the CT hamster and SARS-CoV-2 hamsters on days 3, 7, and 14. SARS-CoV-2, severe acute respiratory syndrome coronavirus 2.

To confirm that SARS-CoV-2 infection was established, we preliminary evaluated the presence of the virus in the noses and lungs of the SARS-CoV-2-infected hamsters on day 3 after oral inoculation with immunohistochemistry and RT-qPCR. The virus was identified in the lungs by immunohistochemistry and RT-qPCR and in the noses by only immunohistochemistry. Viruses were found in various areas of the nasal cavity but were not evenly spread throughout the nasal mucosa. On day 7 post infection, the presence of virus diminished in both the noses and lungs, and the viral load in the lungs was reduced to less than 1/1000th of the Day 3 viral load according to the qPCR analyses. In the nasal cavity, the virus was rarely observed in the area near the nasal septum, but the virus was still present in the outer region. On the 14th day of infection, no virus was identified histologically in either the noses or lungs (**Figures 2A, B**) and no viral genes could be detected with qPCR (**Figure 2C**).

Taken together, in the virus-direct inoculation model, the infection findings were severe on approximately day 3, and the viral load decreased on day 7 after virus administration; by day 14, the virus was almost completely cleared from the nose and lungs (**Figure 2**).

### No SARS-CoV-2 infection signs appeared in the first few days after short-term close contact but became apparent after a certain period following contact

**Figure 3:**
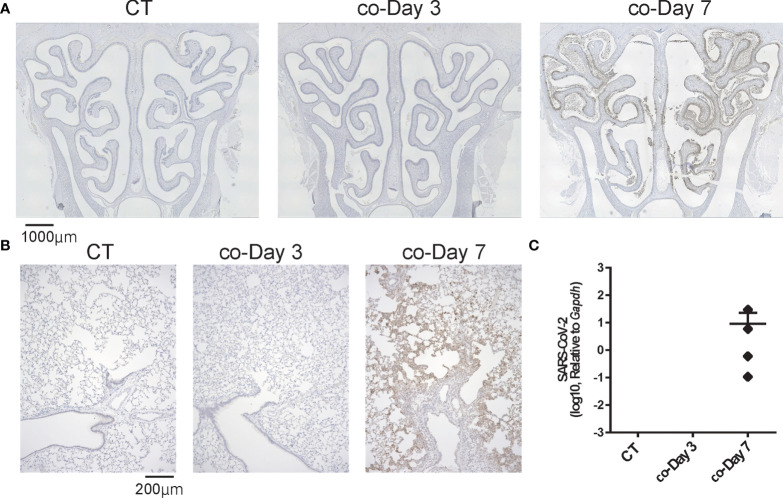
Representative SARS-CoV-2 infection findings in the nose and lungs after short-term close contact with SARS-CoV-2 infected animals. **(A, B)** Representative images of SARS-CoV-2 staining in the nose **(A)** and lungs **(B)** 3 and 7 days after short-term close contact with SARS-CoV-2 infected animals (co-Day 3, co-Day 7). The number of each type of cell in a 300-μm region of each area was counted in the right and left sides of each sample (n = 4, each group). **(C)** SARS-CoV-2 gene detection with RT- quantitative polymerase chain reaction in the CT hamster and contact hamsters on days 3 and 7. SARS-CoV-2, severe acute respiratory syndrome coronavirus 2.

Next, we examined whether SARS-CoV-2 could infect the nose and lungs over time by brief contact with infected animals. SARS-CoV-2 was not identified in the noses or lungs on day 3 after short-term contact, but the virus was extensively identified in both the nose and lungs of all hamsters on day 7 after close contact. In the nasal cavity, the virus was more apparent in the LT area than in the NS area. As for the lungs, on day 7, greater viral load was present in the close-contact group than in the direct-infection group, at an almost similar level to that of day 3 in the direct-infection group (**Figure 3**). The period for the virus to be identified in the tissues was markedly delayed compared to the direct-infection group.

### Transoral SARS-CoV-2 infection influences the ORNs at various differentiation stages over time

**Figure 4:**
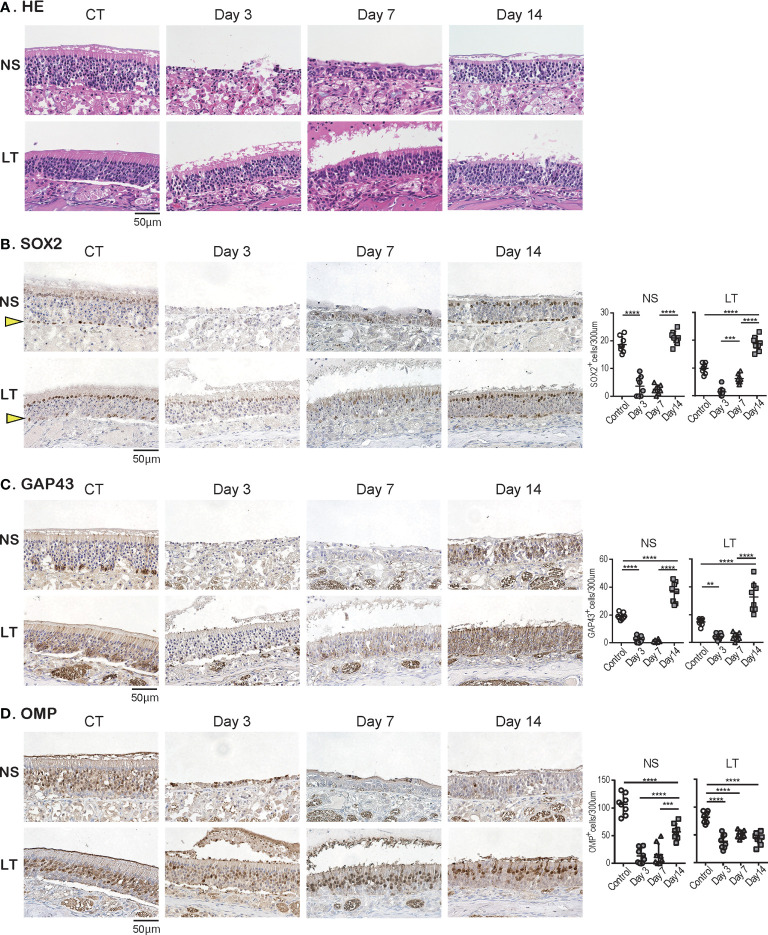
Effects of SARS-CoV-2 infection on the olfactory receptor neuron lineage. **(A)** Representative hematoxylin and eosin staining (HE) images of the olfactory epithelium in control (CT) hamsters and SARS-CoV-2 hamsters on days 3, 7, and 14.**(B-D)**: Representative images of immunohistological staining in CT and SARS-CoV-2 hamsters. Nasal septum (NS) area and lateral turbinate (LT) area are shown in magnified view. Sex-determining region Y-box 2 (SOX2)^+^ progenitor cells **(B)**, growth-associated protein 4 (GAP43)^+^ immature olfactory receptor neurons (ORNs) **(C)**, and olfactory marker protein (OMP)^+^ ORNs **(D)** are shown in brown. The basal layer is indicated by arrows. Tissue sections were counterstained with the nuclear dye hematoxylin (blue). Numbers of SOX2^+^ ORN progenitors per 300 μm of the basal layer, and GAP43^+^ immature ORNs and OMP^+^ mature ORNs per 300 μm of olfactory epithelium in each area are counted in CT or SARS-CoV-2 hamsters. **P < 0.01; ***P < 0.001; ****P < 0.0001. SARS-CoV-2, severe acute respiratory syndrome coronavirus 2.

Subsequently, we examined the temporal effects of SARS-CoV-2 infection on the ORN lineage in the direct-infection model. We found differences in tissue damage between the NS and LT areas; in the NS area, on day 3, almost all superficial cells above the basal layer were missing, especially in the severely damaged areas, but the cellular structure of the OE tended to return to a normal level on day 14 of infection, suggesting OE regeneration. Conversely, in the LT area, the OE did not detach from the basal layer during days 3 to 14 after virus administration (**Figure 4A**). **Figure 4** illustrates representative findings in the most severely affected areas of the OE.

In addition, we examined the impact of SARS-CoV-2 on all ORN lineage cells depending on the OE area. SOX2^+^ olfactory progenitors were scarcely identified in the NS area on days 3 and 7 but recovered to the same level as that in the control group on day 14. In contrast, in the LT area, the number of SOX2^+^ cells decreased on days 3 and 7, but it became higher on day 14 compared to that of the control group (**Figure 4B**). GAP43^+^ immature ORNs could scarcely be seen in both the NS and LT areas on days 3 and 7, whereas the number of these cells increased on day 14 compared to the control group (**Figure 4C**). OMP^+^ ORNs could rarely be observed in the NS area on days 3 and 7, but a recovery tendency in cell counts was evident on day 14, but did not reach the level in the control group. In the LT area, OMR^+^ ORNs existed to some extent in the OE on days 3, 7, and 14, although they were fewer than those in the control group (**Figure 4D**). Thus, the ORN lineage was differently affected by SARS-CoV-2 infection in the NS and LT areas.

### Short-term close contact with SARS-CoV-2 infected models causes late damage in all ORN lineage cells

**Figure 5:**
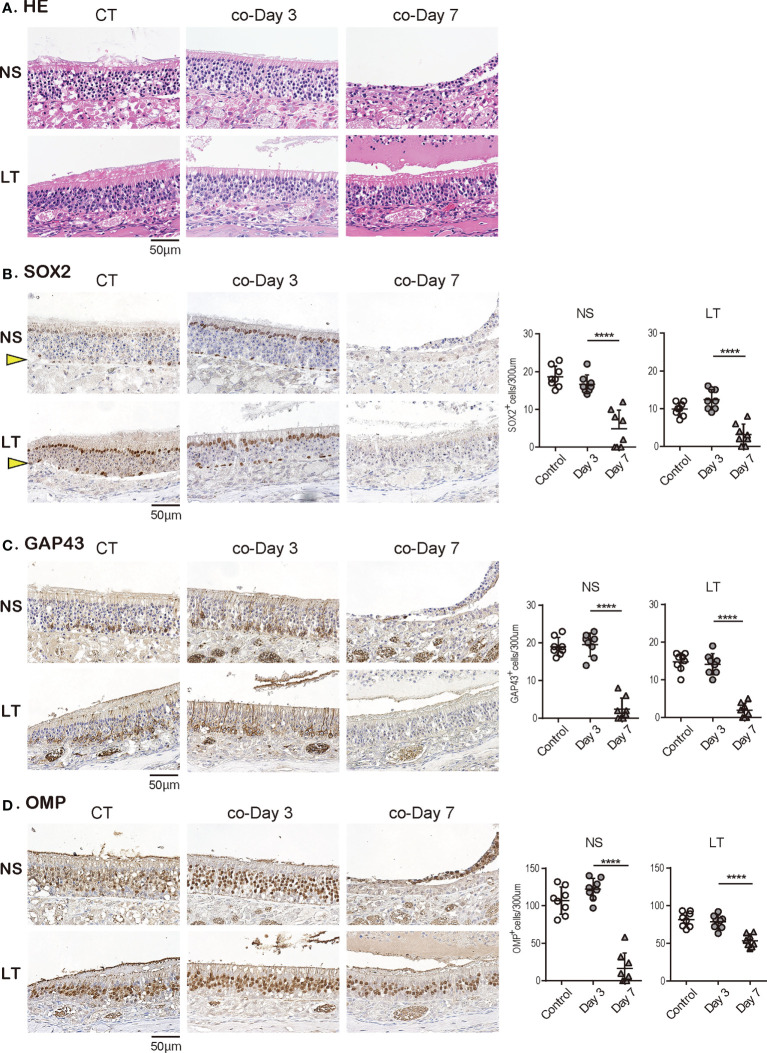
Effects of SARS-CoV-2 on the olfactory receptor neuron lineage after short-term close contact. **(A)** Representative hematoxylin and eosin staining **(HE)** images of the olfactory epithelium in control (CT) hamsters and contact hamsters on days 3 and 7 (co-Day 3, co-Day7).**(B–D)**: Representative images of immunohistological staining. The nasal septum (NS) area and lateral turbinate (LT) area are shown in magnified view. Sex-determining region Y-box 2 (SOX2)^+^ progenitor cells **(B)**, growth-associated protein 4 (GAP43)^+^ immature olfactory receptor neurons (ORNs) **(C)**, and olfactory marker protein (OMP)^+^ ORNs **(D)** are shown in brown. The basal layer is indicated by arrows. Tissue sections were counterstained with the nuclear dye hematoxylin (blue). Numbers of SOX2^+^ ORN progenitors per 300 μm of the basal layer and GAP43^+^ immature ORNs and OMP^+^ mature ORNs per 300 μm of olfactory epithelium in each area are counted in CT or contact hamsters. ****P < 0.0001. SARS-CoV-2, severe acute respiratory syndrome coronavirus 2.

Last, we investigated how late-onset SARS-CoV-2 infection by short-term close contact with infected animals influences the OE over time. Although no structural changes in the OE were observed on day 3 after short-term close contact, marked disruption of the OE in the NS area was observed on day 7 (**Figure 5A**). Immunohistological examination showed no significant differences in the number of all ORN lineage cells of the OE on the 3rd day after contact compared with the control group, which supported the observation that no virus was identified in the tissue. Conversely, in the OE on day 7 after contact, neither SOX2^+^ olfactory progenitors nor GAP43^+^ immature ORNs could be identified in the NS or LT area, with only a few OMP^+^ mature ORNs in the LT area (**Figures 5B-D**). Thus, it was evident that short-term close contact could impair the ORN lineage, and the timing of this impairment was delayed as compared to that by direct virus administration.

## Discussion

**Figure 6:**
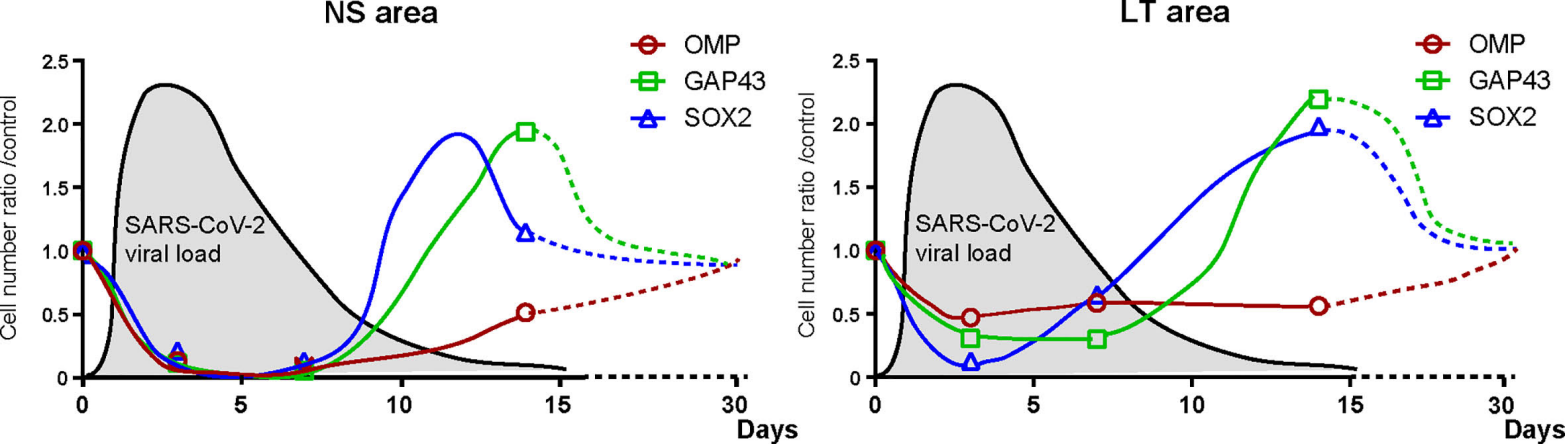
Hypothesis of temporal changes in the SARS-CoV-2 viral load in the lungs and the number of olfactory receptor neuron-related cells in different nasal areas. Changes over time in the ratio of the number of olfactory receptor neuron lineage cells divided by that of the control group at each time point are displayed in the graph. The dashed line shows the speculated future trend. In addition, the estimated viral load of SARS-CoV-2 in the lungs is overlaid on each graph. NS, nasal septum; LT, lateral turbinate; OMP, OMP^+^ mature olfactory receptor neurons; GAP43, GAP43^+^ immature olfactory receptor neurons; SARS-CoV-2, severe acute respiratory syndrome coronavirus 2; SOX2, SOX2^+^ olfactory progenitors.

The present study showed that short-term close contact with infected hamsters did not cause nasal or pulmonary damage by day 3 but resulted in widespread infection of the nose and lungs within 7 days. In contrast, direct SARS-CoV-2 administration caused tissue damage in the nose and lungs with the virus being detected within 3 days. Thereafter, the viral load in the tissues decreased over time, and no virus was identified in the nose or lungs 14 days post infection. We also demonstrated that SARS-CoV-2 extensively damaged the OE, and the degree of OE damage over time varied depending on the OE site. The numbers of ORN-related cells were reduced in all lineage cells with time and then tended to recover (**Figure 6**).

Clinically, the incubation period, required for COVID-19 symptoms to appear, is approximately 6 days (Backer et al., 2020; Cheng et al., 2021), and SARS-CoV-2 RNA shedding lasts approximately 17 days in the upper respiratory tract (Fontana et al., 2021; Cevik et al., 2021). Animal studies using SARS-CoV-2 infection models have reported that the viral load is highest on days 2–5 after virus administration (Lee et al., 2020; Urata et al., 2021; Reyna et al., 2022) and that the virus is eliminated from the tissues on days 8–10 after administration (Urata et al., 2021; Reyna et al., 2022); the direct-infection model in the present study showed a similar trend in viral load. In this study, the SARS-CoV-2 viral load in the contact model hamsters increased after 1 week, despite a short contact time of 30 minutes. Therefore, there is a risk of viral transmission when in contact with SARS-CoV-2 infected individuals, even if it is only for a short period of time (approximately 30 minutes). Regarding viral loads that the short-term contact group and the direct infection group were exposed to, we speculate that the direct infection group was exposed to a higher viral load, resulting in a difference in the time course of infection in the two groups. Even though the virus could not be identified in the nose or lungs on the 3rd day after close contact, severe damage in both tissues was observed on the 7th day after contact, suggesting that the 4th to 6th day after contact with the infected animal was the time when the virus, which had remained latent in the meantime, multiplied. Although the present validation could only examine a period of 7 days after close contact, on day 7, the viral load in the lungs of the animals in the close-contact group was higher than that of the animals in the direct-infection group, implying that another week or longer may be required for the viral load level to decrease to an undetectable level. Temporal changes in viral load and tissue damage after close contact remain a future topic of investigation.

Regarding the transmission form in this short-term close-contact model, as the mouths and noses of the hamsters were not covered, various routes of infection may be considered, including contact, droplet, and airborne infection. Accordingly, if someone infected with SARS-CoV-2 talks or eats with others without a mask for 30 minutes, he or she can spread the infection to others. The percentage of people contracting SARS-CoV-2 and being asymptomatic is estimated to range from 1.2 to 12.9% (Al-Sadeq and Nasrallah, 2020; Jabs et al., 2022). It has now been more than 2 years since COVID-19 became endemic, and the spread of infection has not been well-controlled. If asymptomatic carriers do not take appropriate infection control measures, they may spread the SARS-CoV-2 infection without realizing it.

Olfactory dysfunction caused by SARS-CoV-2 is quite common, and it is now well established how the virus spreads to various olfactory-related tissues, such as the olfactory mucosa, olfactory bulb, and olfactory cortex (Jiao et al., 2021; Vidal et al., 2022; Yu et al., 2022). Regarding the effects of SARS-CoV-2 on the OE, it has been reported that the degree of epithelial damage and recovery rate of the OE differ depending on the location in the nasal cavity (Urata et al., 2021). However, the impact of SARS-CoV-2 on each ORN lineage cell has not been sufficiently verified. We previously reported that all ORN lineage cells are impaired by SARS-CoV-2 (Ueha et al., 2022), and in this study, we verified related longitudinal effects for the first time to our knowledge.

In the NS area, SARS-CoV-2 infection caused shedding of almost all cells of the OE and reduced the width of the OE. The supporting cells of the OE express high levels of ACE2 (Brann et al., 2020; Seo et al., 2021; Ueha et al., 2021), which may be susceptible to infection by SARS-CoV-2. It is possible that SARS-CoV-2 infection of the supporting cells interferes with their structural support, resulting in widespread epithelial shedding. ACE2 is also expressed in the basal olfactory progenitor cells (Brann et al., 2020; Butowt and von Bartheld, 2021), and the low number of SOX2-positive cells on day 3 post infection suggests that SARS-CoV-2 infection may have directly impaired the olfactory progenitor cells. In addition, as the other receptor of SARS-CoV-2, neuropilin-1 is expressed in almost all cells of the OE (Cantuti-Castelvetri et al., 2020; Ziuzia-Januszewska and Januszewski, 2022), it is not surprising that all ORN lineage cells could be affected by SARS-CoV-2. Thereafter, by day 14, the olfactory progenitors and immature ORNs had developed, and epithelial regeneration had become active, but mature ORNs had not sufficiently regenerated, suggesting that overall recovery from SARS-CoV-2 infection-induced OE damage may require longer than 14 days (**Figure 6**). In fact, Urata et al. (2021) and Reyna et al. (2022) reported that in the NS, more than 21 days are needed for the OE thickness to recover to normal. Based on the results of this validation and previous reports, our hypothesis regarding the temporal impact of SARS-CoV-2 on the ORN lineage is presented in **Figure 6**.

In the LT area, the number of OMP-positive cells did not considerably change over time, possibly because some ORNs are impaired by the virus, whereas the unimpaired ORNs remain present in the OE. Thus, it is conceivable that the SARS-CoV-2 receptor expression of the cells in that area may differ depending on the olfactory epithelium site. Moreover, given the increase in GAP43^+^ immature ORNs in the LT area on day 14, the number of impaired OMP^+^ mature ORNs is expected to increase on day 14 or later. As shown in **Figure 6**, the recovery of mature OMPs may need longer in the LT area than in the NS area because of the later timing of the numerical peak of ORN progenitors and immature ORNs in the LT area than in the NS area. Future studies are needed for further observations of the long-term infectious course after short-term close contact.

In conclusion, the present study demonstrated that SARS-CoV-2 can be transmitted even after brief contact and that subsequent OE damage occurs more than 3 days after the trigger of infection. Moreover, SARS-CoV-2 could damage the olfactory receptor system, but the damage could begin to recover approximately 14 days post infection. For SARS-CoV-2 infection control, it is desirable to have a global discussion on the infection control measures that should be implemented and to create common worldwide rules.

## Data availability statement

The raw data supporting the conclusions of this article will be made available by the authors, without undue reservation.

## Ethics statement

The animal experimental protocols followed the ARRIVE guidelines and were approved by The Animal Care and Use Committee at Nara Medical University (approval number, 12922). All procedures were performed in compliance with relevant guidelines on the Care and Use of Laboratory Animals, Nara Medical University and Animal Research, and Animal Care and Use Committee of the University of Tokyo.

## Author contributions

RU developed the concept, designed and performed the experiments, analyzed the data, produced the figures, and wrote the initial draft of the manuscript. TI developed the concept, prepared the animal models, performed some of the experiments, and analyzed the data. SU developed the concept, designed the experiments, and revised the manuscript. RF, MK, and NO-S prepared the animal models, performed some of the experiments, and analyzed the data. TU, HT, and HN performed some of the experiments and analyzed the data. KK and TY developed the concept and critically revised the manuscript. All authors contributed to interpretation of the data and writing of the manuscript.

## Funding

This work was supported by JSPS KAKENHI Grant-in-Aid for Scientific Research (C) [grant number 19K09841], and MSD Life Science Foundation.

## Conflict of interest

The authors declare that the research was conducted in the absence of any commercial or financial relationships that could be construed as a potential conflict of interest.

## Publisher’s note

All claims expressed in this article are solely those of the authors and do not necessarily represent those of their affiliated organizations, or those of the publisher, the editors and the reviewers. Any product that may be evaluated in this article, or claim that may be made by its manufacturer, is not guaranteed or endorsed by the publisher.

## References

[B1] Al-Sadeq D. W. Nasrallah G. K. (2020). The incidence of the novel coronavirus SARS-CoV-2 among asymptomatic patients: A systematic review. Int. J. Infect. Dis. 98, 372–380. doi: 10.1016/j.ijid.2020.06.098 32623083 PMC7330573

[B2] Prevention CDC 2022 COVID-19, appendices. Available at: https://www.cdc.gov/coronavirus/2019-ncov/php/contact-tracing/contact-tracing-plan/appendix.html.

[B3] Backer J. A. Klinkenberg D. Wallinga J. (2020). Incubation period of 2019 novel coronavirus (2019-nCoV) infections among travellers from wuhan, China, 20-28 January 2020. Euro surveillance Bull. Europeen sur les maladies transmissibles = Eur. communicable Dis. Bull. 25 (5). doi: 10.2807/1560-7917.ES.2020.25.5.2000062 PMC7014672 32046819

[B4] Biadsee A. Biadsee A. Kassem F. Dagan O. Masarwa S. Ormianer Z. (2020). Olfactory and oral manifestations of COVID-19: Sex-related symptoms-a potential pathway to early diagnosis. Otolaryngol–head Neck Surg. 163 (4), 722–728. doi: 10.1177/0194599820934380 32539587 PMC7298562

[B5] Boscolo-Rizzo P. Tirelli G. Meloni P. Hopkins C. Madeddu G. De Vito A.. (2022) Coronavirus disease 2019 (COVID-19)-related smell and taste impairment with widespread diffusion of severe acute respiratory syndrome-coronavirus-2 (SARS-CoV-2) omicron variant. Int. Forum Allergy Rhinol. 12(10), 1273–1281. doi: 10.1002/alr.22995 PMC9082058 35286777

[B6] Brann D. H. Tsukahara T. Weinreb C. Lipovsek M. Van den Berge K. Gong B.. (2020). Non-neuronal expression of SARS-CoV-2 entry genes in the olfactory system suggests mechanisms underlying COVID-19-associated anosmia. Sci. Adv. 6 (31). doi: 10.1126/sciadv.abc5801 PMC10715684 32937591

[B7] Bryche B. St Albin A. Murri S. Lacote S. Pulido C. Ar Gouilh M.. (2020). Massive transient damage of the olfactory epithelium associated with infection of sustentacular cells by SARS-CoV-2 in golden Syrian hamsters. Brain behavior Immun. 89, 579–586. doi: 10.1016/j.bbi.2020.06.032 PMC7332942 32629042

[B8] Butowt R. von Bartheld C. S. (2021). Anosmia in COVID-19: Underlying mechanisms and assessment of an olfactory route to brain infection. Neurosci. Rev. J. bringing neurobiol. Neurol. Psychiatry 27 (6), 582–603. doi: 10.1177/1073858420956905 PMC7488171 32914699

[B9] Cantuti-Castelvetri L. Ojha R. Pedro L. D. Djannatian M. Franz J. Kuivanen S.. (2020). Neuropilin-1 facilitates SARS-CoV-2 cell entry and infectivity. Science 370 (6518), 856–860. doi: 10.1126/science.abd2985 33082293 PMC7857391

[B10] Castagnoli R. Votto M. Licari A. Brambilla I. Bruno R. Perlini S.. (2020). Severe acute respiratory syndrome coronavirus 2 (SARS-CoV-2) infection in children and adolescents: A systematic review. JAMA Pediatr. 174 (9), 882–889. doi: 10.1001/jamapediatrics.2020.1467 32320004

[B11] Cevik M. Tate M. Lloyd O. Maraolo A. E. Schafers J. Ho A. (2021). SARS-CoV-2, SARS-CoV, and MERS-CoV viral load dynamics, duration of viral shedding, and infectiousness: a systematic review and meta-analysis. Lancet Microbe 2 (1), e13–e22. doi: 10.1016/S2666-5247(20)30172-5 33521734 PMC7837230

[B12] Cheng C. Zhang D. Dang D. Geng J. Zhu P. Yuan M.. (2021). The incubation period of COVID-19: a global meta-analysis of 53 studies and a Chinese observation study of 11 545 patients. Infect. Dis. poverty 10 (1), 119. doi: 10.1186/s40249-021-00901-9 34535192 PMC8446477

[B13] Fontana L. M. Villamagna A. H. Sikka M. K. McGregor J. C. (2021). Understanding viral shedding of severe acute respiratory coronavirus virus 2 (SARS-CoV-2): Review of current literature. Infection control Hosp. Epidemiol. 42 (6), 659–668. doi: 10.1017/ice.2020.1273 PMC7691645 33077007

[B14] Furukawa R. Kitabatake M. Ouji-Sageshima N. Suzuki Y. Nakano A. Matsumura Y.. (2021). Persimmon-derived tannin has antiviral effects and reduces the severity of infection and transmission of SARS-CoV-2 in a Syrian hamster model. Sci. Rep. 11 (1), 23695. doi: 10.1038/s41598-021-03149-3 34880383 PMC8654961

[B15] Golden J. W. Cline C. R. Zeng X. Garrison A. R. Carey B. D. Mucker E. M.. (2020). Human angiotensin-converting enzyme 2 transgenic mice infected with SARS-CoV-2 develop severe and fatal respiratory disease. JCI Insight 5 (19). doi: 10.1172/jci.insight.142032 PMC7566707 32841215

[B16] Guan W. J. Ni Z. Y. Hu Y. Liang W. H. Ou C. Q. He J. X.. (2020). Clinical characteristics of coronavirus disease 2019 in China. New Engl. J. Med. 382 (18), 1708–1720. doi: 10.1056/NEJMoa2002032 32109013 PMC7092819

[B17] Gussing F. Bohm S. (2004). NQO1 activity in the main and the accessory olfactory systems correlates with the zonal topography of projection maps. Eur. J. Neurosci. 19 (9), 2511–2518. doi: 10.1111/j.0953-816X.2004.03331.x 15128404

[B18] He X. Lau E. H. Y. Wu P. Deng X. Wang J. Hao X.. (2020). Temporal dynamics in viral shedding and transmissibility of COVID-19. Nat. Med. 26 (5), 672–675. doi: 10.1038/s41591-020-0869-5 32296168

[B19] Imai M. Iwatsuki-Horimoto K. Hatta M. Loeber S. Halfmann P. J. Nakajima N.. (2020). Syrian Hamsters as a small animal model for SARS-CoV-2 infection and countermeasure development. Proc. Natl. Acad. Sci. United States America 117 (28), 16587–16595. doi: 10.1073/pnas.2009799117 PMC7368255 32571934

[B20] Imamura F. Hasegawa-Ishii S. (2016). Environmental toxicants-induced immune responses in the olfactory mucosa. Front. Immunol. 7. doi: 10.3389/fimmu.2016.00475 PMC5095454 27867383

[B21] Jabs J. M. Schwabe A. Wollkopf A. D. Gebel B. Stadelmaier J. Erdmann S.. (2022). The role of routine SARS-CoV-2 screening of healthcare-workers in acute care hospitals in 2020: a systematic review and meta-analysis. BMC Infect. Dis. 22 (1), 587. doi: 10.1186/s12879-022-07554-5 35780088 PMC9250183

[B22] Jiao L. Yang Y. Yu W. Zhao Y. Long H. Gao J.. (2021). The olfactory route is a potential way for SARS-CoV-2 to invade the central nervous system of rhesus monkeys. Signal transduction targeted Ther. 6 (1), 169. doi: 10.1038/s41392-021-00591-7 PMC8065334 33895780

[B23] Kishimoto-Urata M. Urata S. Kagoya R. Imamura F. Nagayama S. Reyna R. A.. (2022). Prolonged and extended impacts of SARS-CoV-2 on the olfactory neurocircuit. Sci. Rep. 12 (1), 5728. doi: 10.1038/s41598-022-09731-7 35388072 PMC8987081

[B24] Lauer S. A. Grantz K. H. Bi Q. Jones F. K. Zheng Q. Meredith H. R.. (2020). The incubation period of coronavirus disease 2019 (COVID-19) from publicly reported confirmed cases: Estimation and application. Ann. Internal Med. 172 (9), 577–582. doi: 10.7326/M20-0504 32150748 PMC7081172

[B25] Lee C. Y. Lowen A. C. (2021). Animal models for SARS-CoV-2. Curr. Opin. Virol. 48, 73–81. doi: 10.1016/j.coviro.2021.03.009 33906125 PMC8023231

[B26] Lee A. C. Zhang A. J. Chan J. F. Li C. Fan Z. Liu F.. (2020). Oral SARS-CoV-2 inoculation establishes subclinical respiratory infection with virus shedding in golden Syrian hamsters. Cell Rep. Med. 1 (7), 100121. doi: 10.1016/j.xcrm.2020.100121 32984855 PMC7508015

[B27] Mori K. von Campenhause H. Yoshihara Y. (2000). Zonal organization of the mammalian main and accessory olfactory systems. Philos. Trans. R. Soc. London Ser. B Biol. Sci. 355 (1404), 1801–1812. doi: 10.1098/rstb.2000.0736 11205342 PMC1692907

[B28] Nikolai L. A. Meyer C. G. Kremsner P. G. Velavan T. P. (2020). Asymptomatic SARS coronavirus 2 infection: Invisible yet invincible. Int. J. Infect. Dis. 100, 112–116. doi: 10.1016/j.ijid.2020.08.076 32891737 PMC7470698

[B29] Oran D. P. Topol E. J. (2020). Prevalence of asymptomatic SARS-CoV-2 infection : A narrative review. Ann. Internal Med. 173 (5), 362–367. doi: 10.7326/M20-3012 32491919 PMC7281624

[B30] Organization WH 2022 Coronavirus disease (COVID-19) advice for the public. Available at: https://www.who.int/emergencies/diseases/novel-coronavirus-2019/advice-for-public.

[B31] Reyna R. A. Kishimoto-Urata M. Urata S. Makishima T. Paessler S. Maruyama J. (2022). Recovery of anosmia in hamsters infected with SARS-CoV-2 is correlated with repair of the olfactory epithelium. Sci. Rep. 12 (1), 628. doi: 10.1038/s41598-021-04622-9 35022504 PMC8755745

[B32] Saniasiaya J. Islam M. A. Abdullah B. (2021). Prevalence of olfactory dysfunction in coronavirus disease 2019 (COVID-19): A meta-analysis of 27,492 patients. Laryngoscope 131 (4), 865–878. doi: 10.1002/lary.29286 33219539 PMC7753439

[B33] Seo J. S. Yoon S. W. Hwang S. H. Nam S. M. Nahm S. S. Jeong J. H.. (2021). The microvillar and solitary chemosensory cells as the novel targets of infection of SARS-CoV-2 in Syrian golden hamsters. Viruses 13 (8). doi: 10.3390/v13081653 PMC8402700 34452517

[B34] Tuerdi A. Kikuta S. Kinoshita M. Kamogashira T. Kondo K. Iwasaki S.. (2018). Dorsal-zone-specific reduction of sensory neuron density in the olfactory epithelium following long-term exercise or caloric restriction. Sci. Rep. 8 (1), 17300. doi: 10.1038/s41598-018-35607-w 30470811 PMC6251928

[B35] Ueha R. Ito T. Furukawa R. Kitabatake M. Ouji-Sageshima N. Ueha S.. (2022). Oral SARS-CoV-2 inoculation causes nasal viral infection leading to olfactory bulb infection: An experimental study. Front. Cell. infection Microbiol. 12. doi: 10.3389/fcimb.2022.924725 PMC9234459 35770069

[B36] Ueha R. Kondo K. Kagoya R. Shichino S. Shichino S. Yamasoba T. (2021). ACE2, TMPRSS2, and furin expression in the nose and olfactory bulb in mice and humans. Rhinology 59 (1), 105–109. doi: 10.4193/Rhin20.324 33249429

[B37] Ueha R. Kondo K. Ueha S. Yamasoba T. (2018). Dose-dependent effects of insulin-like growth factor 1 in the aged olfactory epithelium. Front. Aging Neurosci. 10. doi: 10.3389/fnagi.2018.00385 PMC6256067 30515092

[B38] Ueha R. Ueha S. Kondo K. Kikuta S. Yamasoba T. (2018). Cigarette smoke-induced cell death causes persistent olfactory dysfunction in aged mice. Front. Aging Neurosci. 10. doi: 10.3389/fnagi.2018.00183 PMC6008309 29950987

[B39] Ueha R. Ueha S. Kondo K. Nishijima H. Yamasoba T. (2020). Effects of cigarette smoke on the nasal respiratory and olfactory mucosa in allergic rhinitis mice. Front. Neurosci. 14. doi: 10.3389/fnins.2020.00126 PMC7040099 32132898

[B40] Ueha R. Ueha S. Kondo K. Sakamoto T. Kikuta S. Kanaya K.. (2016). Damage to olfactory progenitor cells is involved in cigarette smoke-induced olfactory dysfunction in mice. Am. J. Pathol. 186 (3), 579–586. doi: 10.1016/j.ajpath.2015.11.009 26806086

[B41] Ueha R. Ueha S. Sakamoto T. Kanaya K. Suzukawa K. Nishijima H.. (2016). Cigarette smoke delays regeneration of the olfactory epithelium in mice. Neurotoxicity Res. 30 (2), 213–224. doi: 10.1007/s12640-016-9617-5 27003941

[B42] Urata S. Maruyama J. Kishimoto-Urata M. Sattler R. A. Cook R. Lin N.. (2021). Regeneration profiles of olfactory epithelium after SARS-CoV-2 infection in golden Syrian hamsters. ACS Chem. Neurosci. 12 (4), 589–595. doi: 10.1021/acschemneuro.0c00649 33522795 PMC7874468

[B43] Vidal E. Lopez-Figueroa C. Rodon J. Perez M. Brustolin M. Cantero G.. (2022). Chronological brain lesions after SARS-CoV-2 infection in hACE2-transgenic mice. Veterinary Pathol. 59 (4), 613–626. doi: 10.1177/03009858211066841 PMC9207990 34955064

[B44] Yoshihara Y. Kawasaki M. Tamada A. Fujita H. Hayashi H. Kagamiyama H.. (1997). OCAM: A new member of the neural cell adhesion molecule family related to zone-to-zone projection of olfactory and vomeronasal axons. J. Neurosci. 17 (15), 5830–5842. doi: 10.1523/JNEUROSCI.17-15-05830.1997 9221781 PMC6573213

[B45] Yu P. Deng W. Bao L. Qu Y. Xu Y. Zhao W.. (2022). Comparative pathology of the nasal epithelium in K18-hACE2 tg mice, hACE2 tg mice, and hamsters infected with SARS-CoV-2. Veterinary Pathol. 59 (4), 602–612. doi: 10.1177/03009858211071016 PMC9208069 35094625

[B46] Ziuzia-Januszewska L. Januszewski M. (2022). Pathogenesis of olfactory disorders in COVID-19. Brain Sci. 12 (4). doi: 10.3390/brainsci12040449 PMC9029941 35447981

